# Behavioral Determinants Potentially Relevant to First-Witness Responses in Prehospital Stroke Care: A COM-B-Based Scoping Review

**DOI:** 10.3390/healthcare14132000

**Published:** 2026-07-06

**Authors:** Keying Xu, Chengxia Wei, Hui Ni, Xinhao Chen, Gendi Lu

**Affiliations:** 1 Graduate School, Shanghai University of Traditional Chinese Medicine, Shanghai 201203, China; 22024685@shutcm.edu.cn; 2 Graduate School, Shanghai University of Medicine & Health Sciences, Shanghai 201318, China; 3 Guangming Traditional Chinese Medicine Hospital of Pudong New Area, Shanghai 201314, China; xia97229@163.com (C.W.); 13062626880@163.com (H.N.); CXH_1997@126.com (X.C.)

**Keywords:** stroke, prehospital care, first witnesses, COM-B model, health behavior, lay responders, emergency medical services

## Abstract

**Objective:** Delays in prehospital stroke care are often influenced by the actions of first witnesses (e.g., family, bystanders). However, evidence on what shapes their responses remains fragmented. This scoping review synthesizes these factors and maps them onto the Capability–Opportunity–Motivation–Behavior (COM-B) model. **Methods:** Following the PRISMA-ScR guidelines, eight international and Chinese databases were searched for studies published between 2019 and 2024. Two reviewers independently screened records and charted data. Because no eligible study directly recruited first witnesses, all included studies focused on stroke patients or the general public. We therefore adopted a conservative interpretive approach: we extracted factors occurring at the stroke scene before professional contact and, where a logical low-inference link existed, interpreted their potential relevance to first-witness behavior. These interpreted determinants were then mapped onto the COM-B framework. **Results:** Forty-eight studies involving approximately 257,000 participants from more than 20 countries were included. Factors identified across the literature covered all domains of the COM-B framework. Physical opportunity was the most frequently coded domain (33/134 codings, 24.6%), followed by psychological capability (26/134, 19.4%) and reflective motivation (22/134, 16.4%). Key barriers included insufficient stroke knowledge, limited access to emergency services, and delayed decision-making due to weak urgency perception. **Conclusions:** This scoping review identified behavioral factors potentially relevant to first-witness response, as interpreted from patient and public evidence. The findings suggest that prehospital stroke delays may be associated with the co-occurrence of limited capability, constrained opportunity, and insufficient motivation. These barriers often coexist and may interact with one another, highlighting the potential value of behavior-informed strategies for improving prehospital stroke response and reducing delay.

## 1. Introduction

Stroke is a leading cause of death and long-term disability worldwide, with its burden continuing to rise alongside population aging and lifestyle changes [1]. Each year, more than 12 million people experience a stroke globally, making it a major contributor to mortality and disability [2]. Because the effectiveness of acute stroke treatment is highly time-dependent, early symptom recognition and timely prehospital response are critical for improving outcomes [3,4,5]. The prehospital phase has therefore become a key determinant of treatment efficiency and delay reduction.

In stroke emergencies, first witnesses—such as family members, colleagues, caregivers, or bystanders—play a pivotal role in recognizing symptoms, deciding to seek help, and initiating first aid. Their prompt actions may be associated with more timely access to acute stroke treatment. However, most studies have concentrated on patients themselves or the general public, leaving little systematic synthesis of first-witness roles in prehospital stroke care [6,7]. This gap hampers the development of targeted educational and behavioral interventions to enhance early response.

To address this gap, this review draws on the Capability–Opportunity–Motivation–Behavior (COM-B) model as an analytical framework [8]. The COM-B model conceptualizes behavior as the result of interactions between individuals’ capability to act, the opportunities provided by their environment, and their motivation to respond. Widely applied in public health and behavior change research, the model offers a structured approach for organizing diverse and fragmented evidence into interpretable behavioral domains [9,10,11]. In the context of prehospital stroke care, applying this framework enables a systematic organization of factors potentially relevant to first-witness responses across different settings and study designs.

Accordingly, this scoping review aims to synthesize available evidence from studies of stroke patients and the general public, and to interpret their findings in terms of potential relevance to first-witness responses in prehospital stroke care. These interpreted factors are then organized within the COM-B framework. We acknowledge that direct evidence from first witnesses is currently lacking; thus, this review serves as an initial behavioral mapping of the problem and a call for targeted primary research. By clarifying key behavioral barriers and patterns as they might apply to witnesses, the review seeks to inform future hypothesis-driven studies and support the development of behavior-informed interventions.

## 2. Methods

### 2.1. Study Design

This study was conducted as a scoping review to map and synthesize the breadth of evidence on factors influencing prehospital stroke response. The review was guided by the methodological framework proposed by Arksey and O’Malley [12] and further refined by Levac et al. [13], which emphasizes clear review objectives, iterative study selection and data-charting processes, and a more analytical approach to evidence synthesis and interpretation. The review is reported in accordance with the PRISMA Extension for Scoping Reviews (PRISMA-ScR). The protocol was registered with PROSPERO in April 2025 (registration number CRD420251041493).

### 2.2. Review Questions

The scoping review was guided by the following questions:

1. What factors influencing prehospital stroke response have been reported in the existing literature?
2. How can these reported factors be interpreted in relation to first-witness responses in the prehospital phase of stroke care?
3. How do the identified factors align with the domains of the COM-B framework?

### 2.3. Information Sources and Search Strategy

A comprehensive literature search was conducted in eight databases: Web of Science, PubMed, Cochrane Library, EMBASE, CNKI, Wanfang Data, VIP, and SinoMed. Studies published between January 2019 and December 2024 were considered eligible. The search strategy combined Medical Subject Headings (MeSH) and free-text terms related to stroke (e.g., cerebrovascular accident, cerebral infarction, intracerebral hemorrhage), prehospital care, and influencing factors, and was adapted to the syntax of each database. Reference lists of relevant reviews were also screened to identify additional studies. The full search strategies are provided in the Supplementary Materials.

The review focused on studies published between 2019 and 2024 because substantial developments in stroke public education campaigns, emergency medical service (EMS) systems, and behavioral intervention approaches have occurred during this period. Restricting the review to recent evidence was intended to capture factors most relevant to contemporary prehospital stroke care and current healthcare contexts.

### 2.4. Study Selection

**Figure 1:**
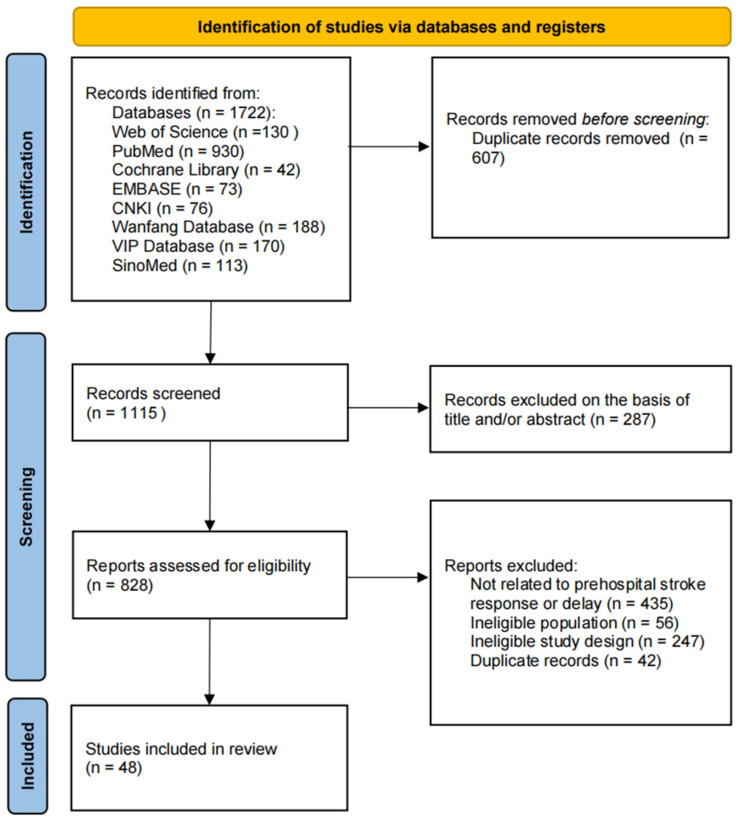
Flowchart for search and inclusion of studies.

All retrieved records were imported into EndNote X9 for management, and duplicate records were removed. Two reviewers (K.X. and C.W.) independently screened titles and abstracts against the eligibility criteria. Full texts of potentially eligible articles were then independently assessed by the same two reviewers. Disagreements at any stage were resolved through discussion, or by consultation with a third reviewer (N.H) when consensus could not be reached. The study selection process is summarized in the PRISMA flow diagram (Figure 1). Inter-rater agreement was substantial (κ = 0.84 for title/abstract screening; κ = 0.91 for full-text assessment).

### 2.5. Eligibility Criteria

Studies were included if they

(1) Used quantitative (cross-sectional, cohort, or case–control), qualitative, or mixed-methods designs;
(2) Examined factors related to prehospital stroke response or delay;
(3) Were published in peer-reviewed journals.

Given the limited number of studies explicitly focusing on first witnesses, studies involving patients or the general public were also included if they reported factors occurring prior to first medical contact and were relevant to prehospital response situations.

Studies were excluded if they lacked full text, were duplicate publications, constituted gray literature (e.g., conference abstracts or study protocols), or did not address factors relevant to prehospital stroke care.

### 2.6. Definition of First Witness for Interpretation

For this review, a first witness is defined as an individual physically present at the stroke scene before EMS arrival, capable of recognizing the event and initiating help. This includes family members, cohabitants, bystanders, and informal caregivers, but excludes patients themselves, remote callers, and professional healthcare providers acting in role. This definition guided our interpretive protocol (Section 2.7): only factors observable or experienceable by such a witness were retained for COM-B mapping.

### 2.7. Data Charting and Identification of Witness-Relevant Factors

Data charting followed a structured form piloted by two reviewers (K.X. and C.W.). Because most included studies recruited patients or the general public rather than first witnesses directly, we applied a conservative interpretive approach to extract witness-relevant factors.

Two reviewers independently examined each reported factor and asked: “Would this factor be directly observable or experienceable by a witness at the stroke scene before EMS arrival?” A factor was retained only if a logical, low-inference link existed. Observable situational factors (e.g., onset time, location) were directly attributed to the witness’s environment; patient-level characteristics (e.g., education) were interpreted as proxies for witness-side challenges only when a plausible mechanism existed. Factors requiring strong assumptions about witness internal states were excluded, including patient-specific clinical conditions such as cognitive impairment, aphasia, loss of consciousness, or stroke severity, which could not reasonably be interpreted as determinants of first-witness response.

All interpretations were documented and reviewed by two reviewers, with disagreements resolved by consensus. This conservative approach prioritizes transparency, acknowledging the lack of direct witness evidence.

### 2.8. COM-B Mapping and Coding Procedures

Drawing on the interpretive approach outlined in Section 2.7, we analyzed the extracted factors through the lens of the COM-B framework, which conceptualizes behavior as the product of interactions between capability, opportunity, and motivation. Rather than directly attributing patient-level characteristics to first witnesses, we situated the analysis at the level of situational context and observable prehospital conditions, including symptom presentation, onset timing, availability of support, and access to emergency services. Two reviewers with expertise in behavioral science (K.X. and C.W.) independently coded the extracted factors into the six COM-B domains: physical capability, psychological capability, physical opportunity, social opportunity, automatic motivation, and reflective motivation. An initial coding framework was piloted on three included studies and refined. Inter-coder reliability was substantial (κ = 0.88). Disagreements were resolved through consensus discussion, with a third reviewer (N.H) consulted when necessary.

To enhance coding consistency, a coding framework based on the COM-B model was developed before formal coding. Coders were instructed to assign factors to the domain representing the most proximal behavioral influence. When a factor could plausibly fit multiple domains, coding decisions were guided by the primary mechanism through which the factor was expected to influence prehospital response. Factors were assigned to multiple domains only when strong conceptual justification existed.

The frequency of factors reported within each domain was calculated to identify patterns and gaps in the literature. This approach allowed for the identification of behaviorally relevant influences while avoiding assumptions of equivalence between patient-level and witness-level attributes.

### 2.9. Data Synthesis

Charted data were synthesized descriptively. Two reviewers independently performed thematic analysis to identify recurring patterns across the extracted factors. Identified themes were then iteratively organized within the COM-B framework through consensus discussion. To support interpretation, identified factors were iteratively organized within the Capability–Opportunity–Motivation–Behavior (COM-B) framework, including physical and psychological capability, physical and social opportunity, and automatic and reflective motivation. This mapping process was used to structure and summarize the range of reported influences within a behavioral framework, rather than to establish causal relationships or compare the relative strength of associations across studies.

## 3. Results

### 3.1. Study Selection

A total of 1722 records were identified across eight databases. After removal of 607 duplicates, 1115 records were screened by title and abstract. Of these, 287 records were excluded, leaving 828 reports for full-text assessment. Following full-text review, 780 reports were excluded for reasons including irrelevant population, outcomes, or study design, resulting in 48 studies being included in the final analysis (Figure 1).

### 3.2. Study Characteristics

The 48 included studies [6,14,15,16,17,18,19,20,21,22,23,24,25,26,27,28,29,30,31,32,33,34,35,36,37,38,39,40,41,42,43,44,45,46,47,48,49,50,51,52,53,54,55,56,57,58,59,60], published between 2019 and 2024, represented more than 20 countries across Asia, Europe, Africa, and the Americas. The largest proportion originated from China (*n* = 26), followed by South Korea (n = 4) and Japan (n = 2), with additional contributions from European countries (e.g., Switzerland, Spain, Denmark, Greece, Portugal), African countries (e.g., Egypt, Morocco, Senegal, Somalia, Congo, South Africa), and the Americas (USA, Mexico). Study designs included cross-sectional surveys (n = 30), retrospective cohort studies (n = 13), prospective cohort studies (n = 4), and one case–control study (n = 1). Sample sizes ranged from 120 to 144,014 participants, with a total of approximately 257,132 participants across all studies. A summary of key study characteristics is presented in Table 1, while detailed extraction information for each included study is provided in Supplementary Table S1.

### 3.3. Interpreted Behavioral Implications for First-Witness Response

Following the interpretive approach outlined in Section 2.7, we analyzed the extracted factors for their relevance to first-witness prehospital behaviors. Although most included studies examined patients or the general public rather than first witnesses directly, the reported influencing factors consistently reflected circumstances occurring before professional medical contact. When translated into a first-witness perspective, these factors converged into several recurring prehospital behavioral challenges (Table 2). These challenges were reflected in impaired symptom recognition, delayed help-seeking decisions, and constraints related to emergency access and transport. Together, these empirically reported behavioral challenges provided the basis for organizing influencing factors within the COM-B framework.

**Table 2:** Interpreted behavioral implications for first-witness response inferred from patient/public evidence.

Category of Influencing Factors	Typical Reported Factors (from Included Studies)	First-Witness Behavioral Challenge	Affected Prehospital Behaviors
Symptom- and knowledge-related factors	Limited awareness of stroke symptoms and therapeutic time window; misinterpretation of atypical or mild symptoms; low health literacy	Potential difficulties in recognizing stroke symptoms and assessing symptom urgency	Symptom recognition; decision-making
Communication-related factors	Non-dominant language use; communication difficulties between patients, witnesses, and emergency services	Potential communication barriers affecting symptom interpretation and help-seeking	Symptom recognition; help-seeking
Environmental and access-related factors	Rural residence; long distance to comprehensive hospitals; limited EMS availability; lack of immediate communication means	Potential constraints related to emergency resource accessibility and care access	EMS activation; choice of care pathway
Temporal and situational factors	Night-time onset; wake-up stroke; symptom discovery outside routine hours	Potential uncertainty regarding symptom onset and perceived urgency	Decision-making; help-seeking
Experiential and contextual factors	Prior stroke experience; presence of multiple comorbidities	Potential influence of prior experiences on symptom appraisal and response decisions	Decision-making; EMS activation

Note: All behavioral implications are inferred from patient/public studies using a conservative interpretive approach (see Section 2.7). This table presents a qualitative synthesis of recurring behavioral challenges derived from the interpretive process. Exact frequencies for each challenge category are reflected in the COM-B codings reported in Table 3.

**Table 3:** COM-B classification of behavioral factors potentially relevant to first-witness stroke response (based on 134 factor codings from 48 studies).

COM-B Model	Core Component	Synthesized Determinants	Examples of Barriers/Facilitators	Number of Factor Codings (N of Studies Reporting at Least One Factor in This Domain)
Capability	Physical	Limited physical health	Frailty, comorbidities affecting reaction speed	20
	Psychological	Insufficient knowledge & skills	Poor awareness of stroke symptoms/time window; misattribution; low health literacy	26
Opportunity	Physical	Limited access to emergency resources	Long transport distance, inadequate EMS coverage; lack of communication means	33
	Social	Insufficient support & communication	Low SES; absence of others at onset, lack of family/community support; language barriers	15
Motivation	Automatic	Weak emergency triggers	Low salience of severity, no habitual “call EMS immediately”, delays due to nocturnal/atypical onset	18
	Reflective	Delayed decision-making	Waiting for spontaneous recovery, preference for primary/village clinics instead of EMS	22

Notes: SES = socioeconomic status; EMS = emergency medical services; Each study could contribute multiple factors across different COM-B domains; therefore, coding frequencies do not represent the number of studies. The frequency column reflects the reporting density of interpreted factors within each domain and should not be interpreted as evidence strength, prevalence, effect size, or relative importance. Percentages may not sum to exactly 100% because of rounding.

### 3.4. Behavioral Factors Potentially Relevant to First-Witness Response Mapped to the COM-B Model

When organized within the COM-B framework, the interpreted factors inferred from patient/public evidence were distributed across all six components potentially relevant to first-witness prehospital stroke response (Table 3). Physical opportunity was the most frequently coded domain, accounting for 33 of the 134 total factor codings (24.6%), followed by psychological capability (26 codings, 19.4%) and reflective motivation (22 codings, 16.4%). Physical capability (20 codings, 14.9%), social opportunity (15 codings, 11.2%), and automatic motivation (18 codings, 13.4%) were also represented. The distribution reflects the reporting density of interpreted factors across COM-B domains. These frequencies should not be interpreted as evidence strength, prevalence, or relative importance of the domains.

## 4. Discussion

### 4.1. Summary of Principal Findings

As a scoping review, this study does not establish causal relationships but synthesizes consistent patterns across heterogeneous evidence. Our findings suggest that first-witness responses, as interpreted from patient/public evidence, may not follow a simple linear progression from symptom recognition to action. Instead, behavior may reflect interactions among capability, opportunity, and motivation within the COM-B framework.

Across the included studies, interpreted factors were distributed across all COM-B domains. Physical opportunity and psychological capability were the domains with the highest reporting density. These findings suggest that barriers to prehospital stroke response may involve both symptom-recognition challenges and contextual constraints.

#### 4.1.1. Capability: Foundational Conditions

Capability may represent an important condition associated with first-witness response. In this review, psychological capability emerged as the dominant limitation, mainly reflected in insufficient knowledge of stroke symptoms and frequent misattribution of atypical presentations such as dizziness or speech disturbance. Consistent with previous research, recognition of non-classic symptoms remains low despite long-term public education efforts [55,61]. Importantly, the findings suggest that knowledge alone may be insufficient to support timely response. Several included studies indicated that factors related to health literacy and confidence in interpreting symptoms may also influence whether stroke recognition is translated into action. Even widely promoted tools such as “Stroke 1-2-0” are not consistently applied in real situations, indicating a gap between awareness and practical use [45]. In contrast, higher educational attainment appears to facilitate better navigation of health information and more timely decision-making [6]. Taken together, these findings suggest that capability-related barriers extend beyond knowledge deficits and may also involve difficulties in applying knowledge in real-world emergency situations.

#### 4.1.2. Opportunity: Environmental Context

Opportunity-related factors accounted for the highest reporting density in this interpretive synthesis. This finding suggests that environmental and social context may be important influences on prehospital stroke response, although the relative strength of these associations cannot be determined from the available evidence. Physical opportunity was frequently constrained by limited emergency medical service (EMS) coverage, long transport times, and inadequate infrastructure, particularly in rural or resource-limited settings [39,44,47,49,59]. Social opportunity also emerged as an important domain. Lower socioeconomic status [7,28,54], lack of family or community support [22,28,45], and language barriers [20] were consistently associated with delayed help-seeking. In the COM-B framework, socioeconomic status was interpreted as a Social Opportunity factor because it reflects access to social and material resources relevant to emergency response. Conversely, the presence of cohabiting family members often facilitated earlier recognition and decision-making, underscoring the protective role of immediate social support [7,54]. These findings suggest that prehospital delay cannot be understood solely at the individual level. Structural conditions and social environments may be important contextual influences on the feasibility and timing of emergency responses.

#### 4.1.3. Motivation: Decision-Making Processes

Motivation may be an important contributor linking recognition and opportunity with emergency response behavior. Both automatic and reflective processes were implicated. Automatic motivation was often weak, as most individuals lack prior experience or rehearsal of stroke emergencies, which may be associated with hesitation rather than immediate response [57,62]. Reflective motivation was also fragile. Some witnesses delayed action by waiting for symptom resolution or consulting primary care facilities, reflecting both limited trust in EMS and insufficient awareness of the time-critical nature of stroke. Similar behavioral patterns have been observed in other acute cardiovascular conditions [63]. These findings suggest that even when capability and opportunity are present, insufficient motivation may still be associated with delayed responses.

### 4.2. Implications for Intervention Design

The findings suggest that future interventions may benefit from moving beyond uniform public education toward more targeted and behavior-oriented strategies.

First, capability can be strengthened through literacy-sensitive [64,65] and scenario-based education [66,67,68,69] rather than one-size-fits-all campaigns. Tailoring interventions by age, educational level, and prior experience may improve relevance and effectiveness. In addition, incorporating practical training methods—such as simulation or repeated drills—may help bridge the gap between knowledge and action [70,71].

Second, addressing opportunity barriers requires system-level improvements. Enhancing EMS accessibility, optimizing dispatch efficiency, and reducing rural–urban disparities are essential for improving timely care [72,73]. At the same time, strengthening community-level support systems, including family engagement and local networks, may facilitate faster decision-making. Beyond these conventional system-level approaches, emerging digital technologies may provide additional opportunities to improve prehospital stroke recognition and response. Dispatcher-assisted telephone triage, AI-supported stroke recognition systems in emergency call centers, and public-access telehealth approaches (e.g., video calls with dispatchers) may facilitate symptom identification, support decision-making, and promote timely EMS activation [73].

Third, motivation can be enhanced by reinforcing both automatic and reflective processes. Repeated exposure to emergency scenarios may help establish habitual responses, while building public trust in EMS and emphasizing the urgency of stroke treatment may support more decisive action [70,71]. Together, these approaches highlight the need for integrated interventions that simultaneously target multiple components of the COM-B system. Future intervention studies should also incorporate measurable process and clinical outcomes to evaluate effectiveness. Potential indicators include symptom-onset-to-call time, EMS activation rates, use of EMS versus private transportation, arrival within the recommended therapeutic window (e.g., 3–4.5 h after symptom onset), and rates of reperfusion treatment, including intravenous thrombolysis and mechanical thrombectomy.

### 4.3. Limitations

This review has several limitations. First, most included studies focused on patients or the general public rather than first witnesses directly. The interpretation of these findings within a first-witness framework may therefore introduce some degree of inference bias, although conservative mapping strategies were applied. Accordingly, the findings should be interpreted as an initial behavioral mapping that is hypothesis-generating rather than confirmatory evidence of first-witness behavior. The extent to which determinants identified in patient- and public-based studies reflect actual first-witness responses remains uncertain and warrants direct investigation in future research. Second, classification of factors within the COM-B model involved subjective judgment, and some determinants may overlap across domains. To mitigate this, independent coding and consensus procedures were used. Third, as a scoping review, this study did not include formal quality appraisal. The included studies varied in design and methodological rigor; therefore, the relative strength of evidence for individual factors cannot be determined. In addition, coding frequency should not be interpreted as evidence strength or relative importance. Furthermore, restricting the search to studies published between 2019 and 2024 may have excluded earlier studies that could provide additional historical or contextual insights into prehospital stroke response. Finally, the geographic distribution of included studies was uneven, with a substantial proportion conducted in China, which may limit generalizability to other settings. Differences in emergency medical service (EMS) organization, healthcare accessibility, public awareness, and sociocultural contexts may influence the applicability of these findings across regions. Although studies from multiple countries were included, the identified COM-B patterns may be more reflective of the settings represented in the current evidence base. Future research should further examine whether these behavioral determinants remain consistent across different healthcare systems and cultural environments. Despite these limitations, the review provides a structured behavioral framework for organizing currently fragmented evidence and generating hypotheses for future research on first-witness responses in prehospital stroke care.

## 5. Conclusions

This scoping review applied the COM-B framework to synthesize behavioral factors potentially relevant to first-witness responses in prehospital stroke care, as inferred from patient and public evidence. The findings indicate that barriers related to capability, opportunity, and motivation commonly co-occur. Physical opportunity was the most frequently coded domain, followed by psychological capability and reflective motivation.

These findings further suggest that emergency-resource accessibility, symptom-recognition capability, and urgency-related decision-making may represent potentially relevant influences on first-witness responses and prehospital delay. Future research should prioritize direct investigation of first witnesses and evaluate behavior-informed interventions across diverse healthcare contexts.

## Figures and Tables

**Table 1:** Summary characteristics of included studies (N = 48).

Characteristic	Categories	Number of Studies (%) or Range
Publication year	2019	14 (29.2%)
	2020	8 (16.7%)
	2021	6 (12.5%)
	2022	9 (18.8%)
	2023	7 (14.6%)
	2024	4 (8.3%)
Geographic region	China	26 (54.2%)
	Other Asia (South Korea, Japan, India, Thailand, Indonesia, Nepal, Saudi Arabia, Iran, Somalia)	12 (25.0%)
	Europe	5 (10.4%)
	Africa	4 (8.3%)
	Americas (USA, Mexico)	1 (2.1%)
Study design	Cross-sectional	30 (62.5%)
	Retrospective cohort	13 (27.1%)
	Prospective cohort	4 (8.3%)
	Case–control	1 (2.1%)
Sample size	Range	120–144,014
	Total participants	257,132
Study population	Stroke patients	44 (91.7%)
	General public/community	3 (6.3%)
	Mixed (patients + physicians)	1 (2.1%)

Note: Detailed extraction of each study, including first author, country, sample size, study population, and specific influencing factors, is provided in Supplementary Table S1.

## Data Availability

No new data were created or analyzed in this study. Data sharing is not applicable to this article.

## Supplementary Materials

The following supporting information can be downloaded at: https://www.mdpi.com/article/10.3390/healthcare14132000/s1, Text S1. Literature search strategies for all databases. Table S1. Characteristics of included studies.
