# Supplementary material for: Behavioral Determinants Potentially Relevant to First-Witness Responses in Prehospital Stroke Care: A COM-B-Based Scoping Review
Source: Healthcare (Basel). 2026 Jul 6;14(13):2000. doi: 10.3390/healthcare14132000 (PMC13361630; doi:10.3390/healthcare14132000)
Supplement: Supplementary file 1 [file healthcare-14-02000-s001.zip › healthcare-4331157-supplementary.pdf]

Supplementary Materials

Supplementary Text S1. Literature search strategies for all databases

Supplementary Table S1: Characteristics of the included studies

Supplementary Text S1. Literature search strategies for all databases

1.Pubmed

Search number	Query
#1	(((((("Stroke"[Mesh]) OR ("Cerebral Infarction"[Mesh])) OR ("Brain Ischemia"[Mesh])) OR ("Ischemic Stroke"[Mesh])) OR ("Embolic Stroke"[Mesh])) OR ("Thrombotic Stroke"[Mesh])) OR ("Hemorrhagic Stroke"[Mesh])
#2	(((((((Strokes[Title/Abstract]) OR (Cerebrovascular Accident[Title/Abstract])) OR (Cerebrovascular Apoplexy[Title/Abstract])) OR (Brain Vascular Accident[Title/Abstract])) OR (Cerebrovascular Stroke[Title/Abstract])) OR (Cerebral Stroke[Title/Abstract])) OR (Subarachnoid Hemorrhagic Strokes[Title/Abstract])) OR (Cerebrovascular Disorders[Title/Abstract])
#3	#1 OR #2
#4	((("Emergency Medical Services"[Mesh]) OR ("First Aid"[Mesh])) OR ("Ambulances"[Mesh])) OR ("Emergencies"[Mesh])
#5	((((((((((((Pre-hospital care[Title/Abstract]) OR (Pre-hospital emergency[Title/Abstract])) OR (prehospital rescue[Title/Abstract])) OR (Mobile first aid facility[Title/Abstract])) OR (Prehospital management[Title/Abstract])) OR (Prehospital delay[Title/Abstract])) OR (Prehospital care[Title/Abstract])) OR (pre-hospital treatment[Title/Abstract] AND care[Title/Abstract])) OR (Pre-Hospital Critical Care[Title/Abstract])) OR (Pre-hospital time[Title/Abstract])) OR (Witness[Title/Abstract])) OR (Bystander[Title/Abstract])) OR (First responder[Title/Abstract])
#6	#4 OR #5
#7	"Risk Factors"[Mesh]
#8	((((((((Risk Factor[Title/Abstract]) OR (associate factors[Title/Abstract])) OR (relevant factors[Title/Abstract])) OR (influence factors[Title/Abstract])) OR (correlated factors[Title/Abstract])) OR (obstructive factor[Title/Abstract])) OR (Barrier factors[Title/Abstract])) OR (stimulative factor[Title/Abstract])) OR (Contributing factors[Title/Abstract])
#9	#7 OR #8
#10	#3 AND #6 AND #9<br>Filters: from 2019-2024

2.Web of Science

Search number	Query
#1	(((((((((((((TS=(Stroke)) OR TS=(Cerebral Infarction)) OR TS=(Brain Ischemia)) OR TS=(Ischemic Stroke)) OR TS=(Embolic Stroke)) OR TS=(Thrombotic Stroke)) OR TS=(Hemorrhagic Stroke)) OR TS=(Strokes)) OR TS=(Cerebrovascular Accident)) OR TS=(Cerebrovascular Apoplexy)) OR TS=(Brain Vascular Accident)) OR TS=(Cerebrovascular Stroke)) OR TS=(Cerebral Stroke)) OR TS=(Subarachnoid Hemorrhagic Strokes)) OR TS=(Cerebrovascular Disorders)
#2	(((((((((((((((TS=(Emergency Medical Services)) OR TS=(First Aid)) OR TS=(Ambulances)) OR TS=(Emergencies)) OR TS=(Pre-hospital care)) OR TS=(Pre-hospital emergency)) OR TS=(prehospital rescue)) OR TS=(Mobile first aid facility)) OR TS=(Prehospital management)) OR TS=(Prehospital delay)) OR TS=(Prehospital care)) OR TS=(pre-hospital treatment and care)) OR TS=(Pre-Hospital Critical Care)) OR TS=(Pre-hospital time)) OR TS=(Witness)) OR TS=(Bystander)) OR TS=(First responder)
#3	((((((((((TS=(Risk Factors)) OR TS=(Risk Factor)) OR TS=(associate factors)) OR TS=(relevant factors)) OR TS=(influence factors)) OR TS=(correlated factors)) OR TS=(obstructive factor)) OR TS=(stimulative factor)) OR TS=(Contributing factors)) OR TS=(Barrier factors)) OR TS=(Hindering factors)
#4	#1 AND #2 AND #3 and 2024 or 2023 or 2022 or 2021 or 2020 or 2019 (Publication Years)

3.Cochrane Library

Search number	Query
#1	MeSH descriptor: [Stroke] explode all trees
#2	MeSH descriptor: [Cerebral Infarction] explode all trees
#3	MeSH descriptor: [Brain Ischemia] explode all trees
#4	MeSH descriptor: [Ischemic Stroke] explode all trees
#5	MeSH descriptor: [Embolic Stroke] explode all trees
#6	MeSH descriptor: [Thrombotic Stroke] explode all trees
#7	MeSH descriptor: [Hemorrhagic Stroke] explode all trees
#8	(Strokes):ti,ab,kw OR (Cerebrovascular Accident):ti,ab,kw OR (Cerebrovascular Apoplexy):ti,ab,kw OR (Brain Vascular Accident):ti,ab,kw OR (Cerebrovascular Stroke):ti,ab,kw
#9	(Cerebral Stroke):ti,ab,kw OR (Subarachnoid Hemorrhagic Strokes):ti,ab,kw OR (Cerebrovascular Disorders):ti,ab,kw
#10	#1 or #2 or #3 or #4 or #5 or #6 or #7 or #8 or #9
#11	MeSH descriptor: [Emergency Medical Services] explode all trees
#12	MeSH descriptor: [First Aid] explode all trees
#13	MeSH descriptor: [Emergencies] explode all trees
#14	(Pre-hospital care):ti,ab,kw OR (Pre-hospital emergency):ti,ab,kw OR (prehospital rescue):ti,ab,kw OR (Mobile first aid facility):ti,ab,kw OR (Prehospital management):ti,ab,kw
#15	(Prehospital delay):ti,ab,kw OR (Prehospital care):ti,ab,kw OR (pre-hospital treatment and care):ti,ab,kw OR (Pre-Hospital Critical Care):ti,ab,kw OR (Pre-hospital time):ti,ab,kw
#16	(Witness):ti,ab,kw OR (Bystander):ti,ab,kw OR (First responder):ti,ab,kw
#17	#11 or #12 or #13 or #14 or #15 or #16
#18	MeSH descriptor: [Risk Factors] explode all trees
#19	(Risk Factor):ti,ab,kw OR (associate factors):ti,ab,kw OR (relevant factors):ti,ab,kw OR (influence factors):ti,ab,kw OR (correlated factors):ti,ab,kw
#20	(obstructive factor):ti,ab,kw OR (stimulative factor):ti,ab,kw OR (Contributing factors):ti,ab,kw OR (Barrier factors):ti,ab,kw OR (Hindering factors):ti,ab,kw
#21	#18 or #19 or #20
#22	#10 and #17 and #21<br>with Publication Year from 2019 to 2024, in Trials

4.EMbase

Search number	Query
#1	'brain ischemia'/exp OR 'ischemic stroke'/exp OR 'cerebrovascular accident'/exp OR 'acute ischemic stroke'/exp
#2	stroke:ab,ti OR strokes:ab,ti OR 'cerebrovascular accident':ab,ti OR 'cerebrovascular apoplexy':ab,ti OR 'brain vascular accident':ab,ti OR 'cerebrovascular stroke':ab,ti OR 'cerebral stroke':ab,ti OR 'subarachnoid hemorrhagic strokes':ab,ti OR 'cerebrovascular disorders':ab,ti
#3	#1 OR #2
#4	'emergency health service'/exp OR 'first aid'/exp OR 'ambulance'/exp OR 'emergency'/exp
#5	'pre-hospital care':ab,ti OR 'pre-hospital emergency':ab,ti OR 'prehospital rescue':ab,ti OR 'mobile first aid facility':ab,ti OR 'prehospital management':ab,ti OR 'prehospital delay':ab,ti OR 'prehospital care':ab,ti OR ('pre-hospital treatment':ab,ti AND care:ab,ti) OR 'pre-hospital critical care':ab,ti OR 'pre-hospital time':ab,ti OR witness:ab,ti OR bystander:ab,ti OR 'first responder':ab,ti
#6	#4 OR #5
#7	'risk factor'/exp
#8	'risk factor':ab,ti OR 'associate factors':ab,ti OR 'relevant factors':ab,ti OR 'influence factors':ab,ti OR 'correlated factors':ab,ti OR 'obstructive factor':ab,ti OR 'stimulative factor':ab,ti OR 'contributing factors':ab,ti OR 'barrier factors':ab,ti OR 'hindering factors':ab,ti
#9	#7 OR #8
#10	#3 AND #6 AND #9
#11	#10 AND (2019:py OR 2020:py OR 2021:py OR 2022:py OR 2023:py OR 2024:py)

5.CNKI

Search number	Query
#1	(Topic: stroke OR apoplexy OR cerebral hemorrhage OR subarachnoid hemorrhage OR cerebral ischemia OR apoplectic OR cerebrovascular accident OR ischemic stroke OR hemorrhagic stroke OR cerebral infarction OR cerebral thrombosis OR lacunar infarction OR transient ischemic attack OR cerebrovascular disease OR thrombotic stroke OR embolic stroke OR cerebral embolism)
#2	(Topic: emergency medical services OR pre-hospital emergency care OR pre-hospital management OR pre-hospital delay OR pre-hospital nursing OR pre-hospital time OR mobile emergency facilities OR ambulance OR first aid OR witness OR first witness OR bystander)
#3	(Topic: influencing factors OR risk factors OR related factors OR barriers OR facilitating factors)
#4	#1 AND #2 AND #3<br>Time range: January 1, 2019 to December 31, 2024

6.Wanfang Database

Search number	Query
#1	(Subject: ("stroke" OR "apoplexy" OR "cerebral hemorrhage" OR "subarachnoid hemorrhage" OR "cerebral ischemia" OR "apoplectic" OR "cerebrovascular accident" OR "ischemic stroke" OR "hemorrhagic stroke" OR "cerebral infarction" OR "cerebral thrombosis" OR "lacunar infarction" OR "transient ischemic attack" OR "cerebrovascular disease" OR "thrombotic stroke" OR "embolic stroke" OR "cerebral embolism") AND Subject: ("emergency medical services" OR "pre-hospital emergency care" OR "pre-hospital management" OR "pre-hospital delay" OR "pre-hospital nursing" OR "pre-hospital time" OR "mobile emergency facilities" OR "ambulance" OR "first aid" OR "witness" OR "first witness" OR "bystander")) AND Subject: ("influencing factors" OR "risk factors" OR "related factors" OR "barriers" OR "facilitating factors")
#2	#1 AND (Year: 2019 OR 2020 OR 2021 OR 2022 OR 2023 OR 2024)

7.VIP Database

Search number	Query
#1	Abstract = stroke OR apoplexy OR cerebral hemorrhage OR subarachnoid hemorrhage OR cerebral ischemia OR apoplectic OR cerebrovascular accident OR ischemic stroke OR hemorrhagic stroke OR cerebral infarction OR cerebral thrombosis OR lacunar infarction OR transient ischemic attack OR cerebrovascular disease OR thrombotic stroke OR embolic stroke OR cerebral embolism
#2	Abstract = emergency medical services OR pre-hospital emergency care OR pre-hospital management OR pre-hospital delay OR pre-hospital nursing OR pre-hospital time OR mobile emergency facilities OR ambulance OR first aid OR witness OR first witness OR bystander
#3	Abstract = influencing factors OR risk factors OR related factors OR barriers OR facilitating factors
#4	#1 AND #2 AND #3
#5	#4 AND Year：2019-2024

8.SinoMed

Search number	Query
#1	(("stroke"[Abstract:Smart] OR "apoplexy"[Abstract:Smart] OR "cerebral hemorrhage"[Abstract:Smart] OR "subarachnoid hemorrhage"[Abstract:Smart] OR "cerebral ischemia"[Abstract:Smart] OR "thrombotic stroke"[Abstract:Smart] OR "embolic stroke"[Abstract:Smart] OR "cerebral embolism"[Abstract:Smart] OR "apoplectic"[Abstract:Smart] OR "cerebrovascular accident"[Abstract:Smart] OR "ischemic stroke"[Abstract:Smart] OR "hemorrhagic stroke"[Abstract:Smart] OR "cerebral infarction"[Abstract:Smart] OR "cerebral thrombosis"[Abstract:Smart] OR "lacunar infarction"[Abstract:Smart] OR "transient ischemic attack"[Abstract:Smart] OR "cerebrovascular disease"[Abstract:Smart]) AND ("emergency medical services"[Abstract:Smart] OR "pre-hospital emergency care"[Abstract:Smart] OR "pre-hospital management"[Abstract:Smart] OR "pre-hospital delay"[Abstract:Smart] OR "pre-hospital nursing"[Abstract:Smart] OR "pre-hospital time"[Abstract:Smart] OR "mobile emergency facilities"[Abstract:Smart] OR "ambulance"[Abstract:Smart] OR "first aid"[Abstract:Smart] OR "witness"[Abstract:Smart] OR "first witness"[Abstract:Smart] OR "bystander"[Abstract:Smart]) AND ("influencing factors"[Abstract:Smart] OR "risk factors"[Abstract:Smart] OR "related factors"[Abstract:Smart] OR "barriers"[Abstract:Smart] OR "facilitating factors"[Abstract:Smart]))
#2	#1 AND 2019–2024[Date]

Table S1: Characteristics of the included studies

First author (year)	Country/region	Sample size (n)	Study population	Study design	Influencing factors
Bhaskar et al. (2019)	Australia	5313	Stroke/TIA patients presenting to ED at a comprehensive stroke centre	Retrospective cohort	Younger age (55–64, 65–74 years); CALD background (Polynesia, South Asia, Mainland Southeast Asia); lower socioeconomic and geographic context; not using state ambulance as mode of arrival
Dimitriou et al. (2019)	Greece	682	Acute ischemic stroke patients admitted to ED	Prospective cohort	Use of emergency medical services (EMS); family history of cardiovascular disease; smoking status (non-smoker); stroke severity at admission (moderately severe vs minor)
Fladt et al. (2019)	Switzerland	336	Patients with diffusion-weighted MRI–confirmed ischemic stroke admitted to a stroke center	Cross-sectional	First contact with family doctor (face-to-face visit); lack of awareness of stroke symptoms; mode of first healthcare professional alerted; transport by emergency medical services (EMS vs non-EMS)
Gonzalez-Aquines et al. (2019)	Mexico	189	Ischemic stroke patients admitted to a university hospital	Prospective cohort	Employment status (being employed); symptom attribution to stroke; perceived severity of stroke symptoms
Nepal et al. (2019)	Nepal	228	Adult ischemic stroke patients presenting to ED	Prospective cohort	Time of onset (daytime vs night); stroke symptom type (facial deviation, speech disturbance); identification of stroke; awareness of stroke treatment; education level (> high school); distance to hospital (<20 km); mode of presentation (direct vs indirect); traffic conditions (heavy traffic); socioeconomic status (income < USD 1000/year); diabetes mellitus
Soto-Cámara et al. (2019)	Spain	322	Patients with stroke symptoms admitted to Burgos University Hospital	Cross-sectional	Immediate help-seeking after symptom onset; time of onset (daytime; weekend); location of onset (outside home); use of prenotification system; perception of inability to control symptoms without assistance; prior knowledge of stroke as a medical emergency; first medical contact via EMS; speech/language difficulties; patient identification of stroke symptoms
Sobral et al. (2019)	Portugal	1247	Acute ischemic stroke patients eligible for thrombolysis	Case–control	Socioeconomic status (beneficiary of social insertion income); access to telephone contact (no phone or landline only); stroke type (posterior circulation stroke); use of prehospital ambulance services
Trent et al. (2019)	USA	351	Stroke patients (adults with AIS presenting to ED)	Cross-sectional	Time of presentation (evening vs daytime); language spoken (non-English: Spanish/other); cerebrovascular risk factors (1–2 or >2 vs 0); rural hospital presentation (vs urban)
Han et al. (2019)	China	156	Acute ischemic stroke patients presenting to the emergency department	Retrospective cohort	Older age; use of emergency medical services (EMS); urban residence; local (non-migrant) residence
Li (2019)	China	210	Acute ischemic stroke patients admitted to the Neurology Department of Xianyang Hospital	Cross-sectional	Time of stroke onset (daytime vs nighttime); first medical contact (primary/secondary hospital); symptom detection by others; awareness of cerebrovascular disease symptoms; mode of transport (EMS vs other); initial symptom characteristics (speech disturbance, visual disturbance, altered consciousness, ≥2 symptoms, limb weakness/numbness, headache)
Mao et al. (2019)	China	469	Acute ischemic stroke patients hospitalized	Retrospective cohort	Payment type (self-paid medical care); living status (living alone); residence (non-urban); wake-up stroke; history of hypertension; age ≥60 years; awareness of urgency of stroke treatment; ambulance transportation; clinical manifestations (language disorder, consciousness disturbance, cognitive impairment); history of atrial fibrillation
Wang et al. (2019)	China	477	Acute stroke patients	Retrospective cohort	Distance from stroke onset location to hospital; absence of consciousness disturbance at onset; stroke type (hemorrhagic vs ischemic); calling the emergency number (120); transport by ambulance (120); awareness of stroke; awareness of being at high risk for stroke

Continued Table S1: Characteristics of the included studies

First author (year)	Country/region	Sample size (n)	Study population	Study design	Influencing factors
Yan (2019)	China	338	Acute ischemic stroke patients	Cross-sectional	Age; residence (rural vs urban); history of cerebral infarction; referral before definitive care; use of emergency medical services (EMS); stroke knowledge level; recognition of stroke symptoms and risk factors; coping style (positive vs negative); distance from onset location to first medical facility
Kielkopf et al. (2020)	Switzerland	1244	Consecutive patients with suspected acute stroke presenting within 24 hours to a prospective hospital registry	Retrospective cohort	Stroke severity (NIHSS score); referral by General Practitioner (GP); self-admission; admission via emergency medical services (EMS); symptom onset during nighttime
Nagao et al. (2020)	Japan	5102	Acute ischemic stroke patients admitted within 24 h of last known well time from a regional multicenter stroke registry	Prospective cohort	Atrial fibrillation; stroke severity (higher NIHSS score); stroke subtype (anterior circulation); immediate detection of symptoms after onset; emergency system use; pre-stroke disability (mRS before onset); onset at home; diabetes; current smoking; dementia; symptom detection during nighttime (00:00–06:00)
Zhu et al. (2020)	China	630	Acute ischemic stroke patients presenting to emergency departments in China	Prospective cohort	Mode of transportation; atrial fibrillation; response to symptoms; speech disturbance; smoking; alcohol consumption; referral presentation; direct visit to hospital after onset; rushing to emergency after onset
Wan et al. (2020)	China	498	Acute ischemic stroke patients	Prospective cohort	Absence of bystanders at symptom onset; non-use of ambulance transport; long distance from onset location to hospital; absence of consciousness disturbance at onset
Wang et al. (2020)	China	447	Stroke patients and physicians participating in a risk-factor survey for healthcare-seeking delay	Cross-sectional	Key risk factors across stages of delay (patient-related delay factors; transport-related delay factors; in-hospital process-related delay factors), including limited patient risk awareness, low use of emergency medical services (EMS/120), and suboptimal care-seeking and transport pathways
Zhou et al. (2020)	China	157	Acute ischemic stroke patients	Retrospective cohort	Initial department visited (lower rate of emergency department presentation); mode of transport (use of public transportation vs ambulance); symptom misattribution to other diseases; non-prominent or unrecognized symptoms; inadequate stroke recognition by patients, family members, or first witnesses; distance from onset location to hospital
Eddelien et al. (2021)	Denmark	479	Patients with acute stroke or transient ischemic attack admitted to or transferred between stroke units	Cross-sectional	First medical contact choice (EMS vs non-EMS); patient-perceived symptom severity (above median); living arrangement (living alone)
Lee et al. (2021)	South Korea	539	Consecutive acute ischemic stroke patients from prospective registries at two tertiary hospitals in Seoul	Cross-sectional	Initial stroke severity (lower NIHSS); pre-stroke disability (higher mRS); sex (female); unclear onset time; education level (≤6 years vs >12 years)
Ghadimi et al. (2021)	Iran	204	Acute ischemic stroke patients admitted to the stroke care unit	Prospective cohort	Consultation after symptom onset; transport by emergency medical services (EMS); patients' perception of AIS symptoms
Kharbach et al. (2021)	Morocco	197	Patients with acute ischemic stroke	Cross-sectional	Illiteracy; patient behavior (waiting for symptoms to disappear; deciding to go directly to hospital); bystander knowledge of stroke as an emergency with a limited therapeutic window; calling an ambulance; distance to hospital (50–100 km); direct admission without referral; presenting symptoms (e.g., vertigo, balance/coordination disturbance)
Wu et al. (2021)	China	402	Acute ischemic stroke patients	Retrospective cohort	Distance from onset location to hospital (>20 km); lack of knowledge about acute ischemic stroke; self-presentation to hospital (non-EMS); referral before definitive care
Nathisuwan (2022)	Indonesia	126	Adult acute ischemic stroke patients treated at three hospitals	Prospective cohort	Wake-up stroke; unawareness of symptom severity; unawareness of stroke symptoms; referral patient (ineffective referral system)

Continued Table S1: Characteristics of the included studies

First author (year)	Country/region	Sample size (n)	Study population	Study design	Influencing factors
Damon et al. (2022)	Senegal	56	Acute ischemic stroke patients (<80 years)	Cross-sectional	Marital status (being married); sex (female); day of onset (weekdays vs weekends); first hospital level visited (level 2–3 vs higher-level centers)
Alkhotani et al. (2022)	Saudi Arabia	98	Acute ischemic stroke patients	Cross-sectional	Low educational level; unemployment status; poor knowledge of stroke
O'Meara et al. (2022)	South Africa	730	Adults with CT-confirmed ischemic stroke	Cross-sectional	Mode of arrival (primary public EMS call vs private/self-transport); access to emergency services; limited stroke education/awareness in a resource-challenged setting
Sheikh Hassan & Yucel (2022)	Somalia	212	Adult patients with acute ischemic stroke	Cross-sectional	Long travel distance (>10 km); transportation via non-ambulance means; living alone; lack of recognition of stroke symptoms; lack of knowledge about thrombolytic treatment; night-time stroke onset; non-hemiplegic presentation
Nasreldein et al. (2022)	Egypt	618	Acute ischemic stroke patients who did not receive IVT, recruited from three university stroke centers	Prospective cohort	Residence (rural vs urban); delayed onset-to-alarm time (OAT); initial misdiagnosis; presentation to non–stroke-ready hospitals; limited availability of stroke-ready facilities
Gao et al. (2022)	China	450	Patients with acute ischemic stroke	Cross-sectional	Rural residence; absence of bystanders at stroke onset; lack of understanding of the urgency of stroke treatment among patients and families; lack of knowledge about thrombolysis and the thrombolysis time window; self-medication and waiting for spontaneous symptom remission
Sun et al. (2022)	China	700	Community residents in Guangdong Province, China, participating in a survey on stroke prehospital delay behavioral intention	Cross-sectional	Age; cohabitation with a partner; awareness of the "Stroke 120" mnemonic; personality traits (openness, emotional stability); perceived social support; negative coping style; insufficient knowledge of stroke
Yuan et al. (2023)	China	78389	Patients with acute ischemic stroke included in the nationwide Bigdata Observatory platform for Stroke of China	Cross-sectional	Age group (young and middle-aged vs ≥65 years); residence (urban vs rural); geographic region and level of regional development
Huang et al. (2023)	China	332	Acute ischemic stroke patients	Retrospective cohort	Mode of arrival (family transport vs emergency services); first medical contact (outpatient clinic); lack of knowledge about stroke; lack of awareness of thrombolysis; lack of awareness of high-risk status
X. Wang et al. (2023)	China	637	Patients with acute ischemic stroke reported to the National Stroke Screening and Prevention Collaborative Platform	Cross-sectional	Rural residence; living alone; symptom onset at night (00:00–05:59) or evening (18:00–23:59); mild initial symptoms; first contact at village clinic or township hospital; poor knowledge of AIS
Y. Wang et al. (2023)	China	1867	Older patients with acute ischemic stroke admitted to three stroke centers in China	Retrospective cohort	Living alone; inter-hospital referral; lack of stroke awareness; use of emergency medical services; stroke severity at onset (NIHSS score); atrial fibrillation; pre-stroke functional status
Wang et al. (2022)	China	502	Patients with ischemic stroke	Retrospective cohort	History of cerebral infarction; altered consciousness at onset; transport by emergency medical services (EMS/120); symptom first detected by bystanders; headache at onset; inter-hospital transfer
Zhong et al. (2022)	China	136	Acute ischemic stroke patients	Retrospective cohort	Lower educational level (high school or below); impaired consciousness; self-presentation to hospital; living alone; rural residence; first-ever stroke; night-time onset
Butdee et al. (2023)	Thailand	120	Older adults with first-time acute ischemic stroke treated at two hospitals	Cross-sectional	Patient-perceived severity of ischemic stroke; distance from home to hospital
Zhong (2023)	China	200	Patients with acute ischemic stroke	Cross-sectional	Place of residence (urban vs rural); time of stroke onset (daytime vs nighttime); presence of impaired consciousness at onset; recognition that symptoms were cerebrovascular in origin; first medical contact (tertiary hospital vs other hospitals); mode of transportation (EMS/120 vs public transport); level of awareness of the "Stroke 120" mnemonic

Continued Table S1: Characteristics of the included studies

First author (year)	Country/region	Sample size (n)	Study population	Study design	Influencing factors
Edakkattil et al. (2024)	India	470	Adult patients with acute ischemic stroke	Cross-sectional	Lack of awareness of stroke symptoms; unavailability of a vehicle at the event site; longer distance from first medical contact to emergency department; socioeconomic status (upper middle class vs lower class)
Jiang & Zhao et al. (2024)	China	1419	Patients with recurrent acute ischemic stroke	Retrospective cohort	Living with others; lack of stroke knowledge; residential status; low awareness of stroke symptoms; presence/absence of conscious disturbance; diabetes mellitus awareness; physical weakness; mode of hospital presentation; stroke subtype; coronary artery disease
Jiang & Xiong et al. (2024)	China	1419	Patients with acute ischemic stroke	Retrospective cohort	Immigrant status; male gender; age group (younger males <45 years, elderly females); self-acknowledged diabetes; small-vessel stroke; wake-up stroke; educational level (high school and above); stroke-related knowledge; level of consciousness (GCS score); conscious disturbance; limb weakness
Kazadi Kabanda et al. (2024)	Congo	202	Patients with acute stroke and/or accompanying family members interviewed	Prospective cohort	Unmarried status; low education level; absence of impaired consciousness; absence of prior hypertension; absence of prior diabetes; heavy alcohol consumption; non-severe stroke presentation; ischemic stroke subtype; low awareness of stroke symptoms
Lee et al. (2024)	South Korea	144014	Patients with acute ischemic stroke or transient ischemic attack included in a nationwide acute stroke registry	Retrospective cohort	Older age (>65 years); female sex; hypertension; diabetes mellitus; smoking; premorbid disability; mild stroke severity; regional inequality in healthcare access
Liao et al. (2024)	China	3459	Patients with acute ischemic stroke prospectively recruited from nine tertiary general hospitals	Cross-sectional	Rural residence; interhospital transfer; distance from onset location to first-visit hospital (>20 km vs ≤20 km); transportation by emergency medical services; history of atrial fibrillation; stroke severity (mild vs moderate/severe)
Zhu et al. (2024)	China	528	Young adults with acute ischemic stroke aged 18–50 years	Retrospective cohort	Younger age subgroup (36–50 vs <36 years); low stroke awareness; TOAST stroke subtype; ambulance arrival vs non-ambulance transport; dysarthria; dizziness; wake-up stroke; baseline disability (mRS on admission)
Wu et al. (2024)	China	230	Patients with acute ischemic stroke	Cross-sectional	Older age; no previous stroke history; absence of prehospital first aid; rural residence; private transportation; traffic congestion; distance from hospital ≥5 km at onset; lack of stroke-related knowledge dissemination
